# From Capsids to Complexes: Expanding the Role of TRIM5α in the Restriction of Divergent RNA Viruses and Elements

**DOI:** 10.3390/v13030446

**Published:** 2021-03-10

**Authors:** Kevin M. Rose, Stephanie J. Spada, Rebecca Broeckel, Kristin L. McNally, Vanessa M. Hirsch, Sonja M. Best, Fadila Bouamr

**Affiliations:** 1 Laboratory of Molecular Microbiology, National Institute of Allergy and Infectious Diseases, National Institutes of Health, Bethesda, Rockville, MD 20894, USA; rosekem@nih.gov (K.M.R.); stephanie.spada@nih.gov (S.J.S.); vhirsch@niaid.nih.gov (V.M.H.); 2 Laboratory of Virology, Rocky Mountain Laboratories, National Institute of Allergy and Infectious Diseases, National Institutes of Health, Hamilton, Montana, MT 59840, USA; rebecca.broeckel@nih.gov (R.B.); mcnallyk@niaid.nih.gov (K.L.M.); sbest@niaid.nih.gov (S.M.B.)

**Keywords:** TRIM5α, flaviviruses, HIV-1, ubiquitin, innate immunity, ribonucleoprotein

## Abstract

An evolutionary arms race has been ongoing between retroviruses and their primate hosts for millions of years. Within the last century, a zoonotic transmission introduced the Human Immunodeficiency Virus (HIV-1), a retrovirus, to the human population that has claimed the lives of millions of individuals and is still infecting over a million people every year. To counteract retroviruses such as this, primates including humans have evolved an innate immune sensor for the retroviral capsid lattice known as TRIM5α. Although the molecular basis for its ability to restrict retroviruses is debated, it is currently accepted that TRIM5α forms higher-order assemblies around the incoming retroviral capsid that are not only disruptive for the virus lifecycle, but also trigger the activation of an antiviral state. More recently, it was discovered that TRIM5α restriction is broader than previously thought because it restricts not only the human retroelement LINE-1, but also the tick-borne flaviviruses, an emergent group of RNA viruses that have vastly different strategies for replication compared to retroviruses. This review focuses on the underlying mechanisms of TRIM5α-mediated restriction of retroelements and flaviviruses and how they differ from the more widely known ability of TRIM5α to restrict retroviruses.

## 1. Introduction

The innate immune system evolved as an intracellular response to a variety of pathogens including viruses, bacteria, and fungi [1]. Well studied facets of this surveillance system include Toll-like receptors (TLRs), RIG-I-like receptors (RLRs), NOD-like receptors (NLR) and the cGAS-STING pathway that recognize pathogen-associated molecular signatures and trigger immune signaling [2]. However, in the case of the Human Immunodeficiency Virus (HIV-1), one of the most potent innate immune sensors and restriction factors to retroviral infection is rhesus macaque TRIM5α (RhTRIM5α) [3]. Although poor in its ability to restrict HIV-1 replication, human TRIM5α (HuTRIM5α) also protects against N-tropic murine leukemia virus (N-MLV) and equine infectious anemia virus (EIAV) [4,5]. TRIM5α is a E3 ubiquitin ligase that belongs to the tripartite motif (TRIM) family of proteins that is recognized to include approximately 100 members, many of which participate in innate immune signaling [6,7]. Each member of this family contains an N-terminal tripartite motif consisting of a RING finger E3 ligase domain, followed by one or two B-box zinc finger domains, and a coiled coil domain. The N-terminal domain is important for enzyme activity, while the latter two cooperatively control subcellular localization and higher order assembly, respectively [8,9]. The TRIM family members diverge at their C-termini by the presence or absence of one or more functional domains including the C-terminal subgroup One Signature (COS) microtubule binding domain (TRIM1), the bromodomain and plant homeodomain (PHD) finger domains that bind to histones (TRIM33), and more commonly, a single B30.2 or PRY-SPRY (SPRY) domain as is found in TRIM5α [10,11,12]. An additional variant of TRIM5α has also been described in rodents and owl monkeys where Cyclophilin A functionally replaces the SPRY domain (TRIM-Cyp) as a result of a Long INterspersed Element 1 (LINE-1) retrotransposition event into the TRIM5α locus [13,14]. The remainder of this review will shed some light on the new mechanism of flavivirus restriction by TRIM5α and compare these details with the mechanisms involved during antiretroviral and retroelement responses.

## 2. Innate Immune Detection of RNA Viruses by Members of the Tripartite Motif (TRIM) Gene Family

**Figure 1:**
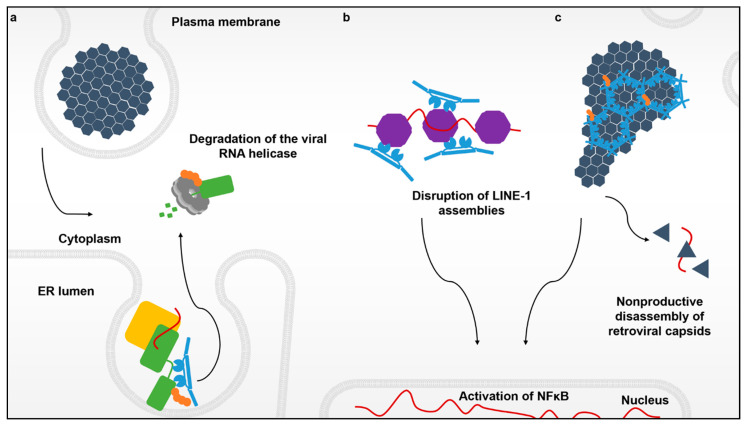
The various interactions between TRIM5α and its targets. Flaviviruses enter the cell cytoplasm via endocytosis and create replication complexes anchored to modified membranes of the endoplasmic reticulum. (**a**) Recognition of the active flavivirus replication complex (yellow) by TRIM5α (blue) that leads to the ubiquitination (orange) and degradation of the viral RNA protease-helicase (green) via the proteasome (light grey). (**b**) Innate immune sensing and disruption of cytoplasmic assemblies of the retroelement LINE-1 (purple). (**c**) The retroviral capsid lattice (black) coated by hexagonal assemblies of TRIM5α. These higher-order TRIM5α oligomers not only induce premature uncoating of the retroviral genome, but also create a platform to promote a TRIM5α-mediated antiviral state via induction of NF-κB signaling.

RhTRIM5α (referred to as TRIM5α from this point forward, unless otherwise noted) was identified as the predominant block to retroviral infection by Simian Immunodeficiency Virus (SIV) in rhesus macaques as evidenced by its ability to limit cross-species transmission and drive the evolution of the retroviral capsid protein [15,16]. The ability of TRIM5α to restrict incoming retroviruses has been attributed to the SPRY domain and its strong positive selection that allows for binding to diverse and evolving retroviral capsid cores [17]. Simultaneously, the B-box and RING domains form higher order hexagonal assemblies around the capsid lattice which prematurely accelerate the uncoating of the viral genome and block the essential step of reverse transcription, while also promoting an antiviral state by stimulating NF-κB signaling [18]. In parallel, the disruption of cytoplasmic ribonucleoprotein (RNP) complexes, and the induction of NF-κB is a recently proposed mechanism for the sensing and restriction of the retroelement LINE-1 by TRIM5α, which functionally also depends on each of its domains [18,19,20,21] (Figure 1b,c). Since the discovery of TRIM5α, numerous additional TRIM family members have been characterized for their ability to also restrict retroviruses. To name a few, the SPRY-less TRIM33 has been shown to degrade the nuclear preintegration complex (PIC) of HIV-1, while TRIM22 disrupts the assembly of transcription machinery required for the newly integrated HIV provirus to propagate itself and make progeny virions [22,23]. However, retroviruses are but one of the many targets of TRIM family members. For instance, TRIM56 restricts both Coronaviruses and Flaviviruses [24]. TRIM25 interacts with foreign single and double stranded RNA (dsRNA), and specifically disrupts Influenza virus replication by binding to viral ribonucleoproteins in the nucleus [25]. TRIM6 has been shown to bind and ubiquitinate the RNA helicase VP35 of Ebola, a filovirus, via its SPRY and RING domains, respectively, but this interaction enhances viral replication [26]. By virtue of the same mechanism, TRIM69 has been shown to restrict Dengue virus (DENV), a mosquito-borne flavivirus, by binding to the viral protease-helicase NS3 and degrading it via the ubiquitin proteasome system [27]. Remarkably, this exact antiviral response was also demonstrated by the potent restriction of several emergent tick-borne flaviviruses by TRIM5α [28] (Figure 1a). This can perhaps be partly explained by the exquisite genetic plasticity of the TRIM5α SPRY domain, allowing for the evolution of a single viral recognition module that targets multiple virus families, including some that are yet to be identified [29].

## 3. Differential Requirements for Ubiquitin in the Restriction and Sensing of RNA Viruses by TRIM5α

The restriction and sensing of RNA viruses by TRIM5α, as well as other TRIM proteins, is intimately linked to ubiquitin signaling due to the conservation of an E3 ubiquitin ligase RING domain present in all of these family members [30]. During infection by HIV-1, TRIM5α multimerizes into hexagonal assemblies that may disrupt incoming capsid cores while simultaneously promoting its E3 ligase activity of forming K63 ubiquitin chains. These same K63 ubiquitin chains also trigger NF-κB signaling, effectively coupling retroviral capsid disassembly with the generation of an antiviral state [20,31]. Although this precise mechanism was not determined for the sensing and restriction of LINE-1 elements by TRIM5α, loss of the RING domain and NF-κB signaling relieves TRIM5α-mediated restriction, hinting at a role for ubiquitin in this process [21]. Interestingly, E3 ligase activity is dispensable for retroviral restriction, suggesting that TRIM5α interference with retroviral replication may involve multiple mechanisms [32,33,34]. However, there is mounting evidence that the E3 ligase activity of TRIM5α may promote the destruction of retroviral proteins by proteasome recruitment or via autophagy, although the latter is highly debated [35,36,37,38]. Indeed, this has been shown to be the case for TRIM33-mediated proteasomal degradation of the HIV preintegration complex (PIC) signaled by conjugation of K48 ubiquitin chains to the retroviral Integrase protein [23]. Nevertheless, the precise role of the RING domain of TRIM5α in retroviral restriction remains to be elucidated.

In stark contrast to the role of ubiquitin in retroviral restriction, a clear role for K48 ubiquitin conjugation by the RING domain of TRIM5α has been described for the select restriction of several tick-borne flaviviruses like tick-borne encephalitis virus (TBEV) and Langat virus (LGTV), but not mosquito-borne flaviviruses like Dengue Virus (DENV) [28]. During tick-borne flavivirus infection, TRIM5α binds to NS3, the viral RNA helicase in complex with its NS2B cofactor (NS2B/3) and promotes its degradation. This mechanism was confirmed via the immunoprecipitation of TRIM5α by NS3, revealing a physical interaction in the cells. Immunofluorescence showed clear perinuclear colocalization between TRIM5α, NS3, and double stranded RNA (dsRNA), a replication intermediate. This suggests that TRIM5α targets sites of active flavivirus replication in the endoplasmic reticulum (ER) through its interactions with NS3. The truncation of the SPRY domain and inactivation of the RING domain of TRIM5α by mutagenesis abolished binding to and K48 ubiquitin conjugation of NS3 in infected cells, respectively. The truncation of NS3 revealed that the viral protease domain and interdomain linker were both necessary and sufficient for binding and degradation by TRIM5α. NS3 abundance could be rescued by treatment with the proteasome inhibitor epoxomicin, but not the lysosome inhibitor bafilomycin A1, supporting a mechanism of proteasomal clearance and not autophagy.

TRIM5α binding to retrovirus capsids is associated with TRIM5α-RING-dependent synthesis of unanchored K63-linked ubiquitin chains that trigger innate immune signaling through TAK1-AP-1/NF-κB activation and expression of cytokines including CXCL10, IL-6, and IL-8 [18,39]. However, it is not known whether interactions between the flavivirus protease and TRIM5α induce NF-κB/AP-1-dependent signal transduction analogous to interactions with the HIV-1 capsid lattice. Therefore, we expressed Rhesus macaque TRIM5α in HEK293 cells to examine AP-1-dependent Firefly luciferase activity. Transfection efficiency was normalized using the constitutively expressed *Renilla* luciferase and dual luciferase assays were performed according to manufacturer’s protocol (Promega). As shown previously [18], rhTRIM5α-stimulated AP-1-dependent luciferase gene expression required a functional RING domain and occurred at levels similar to that induced by mitochondrial antiviral signaling protein (MAVS) used as a positive control. However, rhTRIM5α-dependent reporter gene expression was not further enhanced by coexpression with LGTV NS2B/3 and was instead reduced in a dose-dependent fashion. In addition, mRNA expression for *IFNβ*, *CXCL10* or *IL8* cytokines in LGTV-infected A549 cells was not affected by depletion of *TRIM5* mRNA (the knockout for TRIM5α in these cells in shown in Figure 2 of [28]. Thus, TRIM5α does not function as a pattern recognition receptor for the flavivirus protease to promote cytokine expression. As the RING domain of TRIM5α is required for both K63-linked ubiquitin signaling in cytokine signaling [18] and K48-linked ubiquitination in NS2B/3 degradation [28], it is possible that these ubiquitin conjugation events by TRIM5α cannot be completed simultaneously.

## 4. Recognition of Distinct Viral Ribonucleoproteins in Separate Cellular Compartments

For flaviviruses, replication takes place in ER-derived vesicle packets that shield the viral RNA from detection by host sensors. Viral replicase proteins like NS3 can be localized and sequestered within virus-induced membrane invaginations in a manner that promotes virus replication while shielding the viral genome from cytosolic innate immune sensors like TRIM25 and its associated RIG-I foreign nucleic acid sensor [40,41]. Remarkably, TRIM5α is capable of accessing the cytoplasmic side of these replication organelles and disrupts viral propagation by degrading the viral protease-helicase that unwinds the dsRNA viral replication intermediate (Figure 1a).

Since its discovery over a decade ago, it was believed that the sole function of TRIM5α was to restrict retroviruses like HIV-1. In this scenario, TRIM5α responds to and multimerizes on incoming soluble capsid cores in the cytoplasm (Figure 1c) that contain the active viral reverse transcription complex (RTC) as well as the enzymes for creating the preintegration complex, the two essential viral nucleoprotein complexes for establishing infection. The core itself is in essence, an incredibly large nucleoprotein complex that functions to protect and deliver the viral genome to the host cell nucleus. Surprisingly, TRIM5α was also shown to bind to and sense the RNPs belonging to the retroelement LINE-1, triggering the same antiviral responses as does the retroviral core (Figure 1b) [21].

## 5. Evasion of TRIM5α Surveillance

Although TRIM5α is a potent restriction factor for several pathological RNPs, some of its targets have evolved to evade its immune response. In the case of SIVs, the retroviral capsid protein possesses the genetic plasticity to differentially alter its cytoplasmic surface in order to avoid detection of both TRIM5α and TRIM-Cyp [16]. In parallel, HIV incorporates multiple host proteins into its capsid lattice that prevent recognition by TRIM5α, as is true for Cyclophilin A (CypA), but to also facilitate nuclear entry, as with CPSF6 [42,43]. Indeed, it is now accepted that CypA masks the HIV capsid binding site required for sensing of the retroviral lattice by TRIM5α, thereby attenuating the innate immune response and increasing viral fitness [42,44]. Curiously, Flavivirus polymerases, which are intimately associated with their cognate RNA helicases, have been shown to bind CypA. However, TRIM-Cyp was incapable of restricting flaviviral replication, in opposition to the potency of restriction seen against retroviruses and retroelements [21,28]. Perhaps the incorporation of CypA into flaviviral replication complexes is spatially prohibitive for the RING domain of TRIM5α to conjugate ubiquitin to, and degrade, the viral helicase.

While characterizing the molecular basis for TRIM5α restriction of flaviviruses, we uncovered the surprising ability of NS3 to antagonize TRIM5α-mediated immune activation. Since flaviviral restriction was seen despite the antagonism of this function of TRIM5α, its significance in regard to viral propagation was not pursued. However, there is some evidence to suggest that flaviviruses could dampen the innate immune activation intentionally to promote virus replication. Recently it was shown that TRAF6, a RING E3 ubiquitin ligase like TRIM5α, is a target for degradation by the classic swine fever flavivirus (CSFV) NS3 protease-helicase [45]. TRAF6 inhibits CSFV replication through potent induction of interferon, much like the function of TRIM5α antagonized by tick-borne flaviviruses. Therefore, it seems that one possible explanation for the downregulation of TRIM5α-mediated AP-1 activation by NS3 is to prevent the induction of innate immune signaling. It is noteworthy that patients infected with Hepatitis C Virus (HCV), another flavivirus, also exhibit an impaired cytokine response in addition to reduced levels of TRAF6 [46]. Antagonism of the innate immune activation appears to be a hallmark of flavivirus infection that in some cases is attributable to NS3 [47]. Nevertheless, this activity of NS3 cannot spare tick-borne flaviviral replication sites from disruption by TRIM5α.

## 6. Conclusions

How TRIM5α has evolved to patrol two separate cellular compartments and overcome virus-induced barriers for the restriction of distinct viral and retroelement nucleoproteins remains unclear. Further still, molecular details regarding its protein targets remain obscure. For instance, the ability of mosquito-borne flaviviruses to evade immune detection by TRIM5α despite near complete structural conservation and 50% sequence identity across NS3 proteins, remains unanswered [48]. Additionally, how LINE-1 ORF1 assembles RNPs via multimerization on the LINE-1 genomic RNA and passes through the nuclear envelope, and why these complexes are recognized by the SPRY domain of TRIM5α is also unknown [49]. Given the extremely broad structural and sequence variation between the three protein targets of TRIM5α discussed in this review, it is currently not understood how TRIM5α is poised to encounter each of these pathogens at critical locations and steps in each of their entity’s lifecycles. However, some TRIM proteins are shown to bind RNA [50] and therefore the role of genomic and viral RNAs in these processes needs to be further explored. Regardless, TRIM5α effectively acts as a broad-spectrum antiviral that displays surprising plasticity to disrupt a diverse array of pathological RNPs irrespective of their specific molecular signatures. Taken together, these studies highlight the remarkable ability of a single protein to facilitate an extremely broad range of cellular surveillance mechanisms, which has significant implications for all the other members of the TRIM gene family.

## Figures and Tables

**Figure 2:**
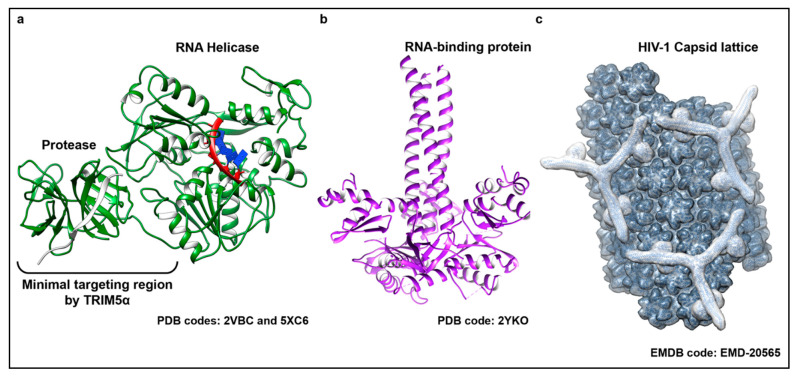
The structurally diverse substrates detected by TRIM5α. TRIM5α has been shown to target and respond to viral proteins belonging to evolutionarily divergent virus families. (**a**) Representative structure of the active RNA helicase protein shared by members of the flavivirus genus. Shown here (colored in green) is a superposition of two crystal structures from the DENV NS3 protein bound to a substrate single stranded RNA (ssRNA) (PDB code: 5XC6) and its protease cofactor NS2B (shown in light grey) (PDB code: 2VBC). The individual domains of the protein and the recognition site for TRIM5α binding are indicated. (**b**) Trimeric ORF1 RNA-binding protein that is the main protein component of LINE-1 RNPs (shown in purple) (PDB code 2YKO). (**c**) Molecular modeling of a hexagonal TRIM5α assembly (outlined in light grey) bound to a lattice of HIV-1 capsid (dark grey) based on an electron density map recently resolved by electron tomography (ET) (EMD-20565). Capsid monomers and TRIM5α dimers and trimers were manually placed into the density using known structures of capsid (PDB code: 3J34) and molecular models, respectively.

## Data Availability

Not applicable.
